# Unilateral Brown Fat FDG Uptake after Childhood Sympathectomy Mimicking Malignancy Resolved by Hybrid PET/MR Image Fusion

**DOI:** 10.1055/s-0045-1812308

**Published:** 2025-10-24

**Authors:** Freimut Dankwart Eberhard Juengling, Judith Hafer, Alin Chirindel

**Affiliations:** 1 Department of Oncology, University of Alberta, Edmonton, Alberta, Canada; 2 Department of Oncology, University Hospital of Basel, Basel, Kanton Basel-Stadt, Basel-Stadt, Switzerland; 3 Department of Nuclear Medicine, University of Basel, Kanton Basel-Stadt, Basel-Stadt, Basel, Switzerland

**Keywords:** brown adipose tissue, Horner syndrome, magnetic resonance imaging, PET/MRI, positron emission tomography, stellate ganglion, sympathectomy

## Abstract

Physiologic ^18^ F-fluorodeoxyglucose (FDG) uptake in brown adipose tissue (BAT) is a recognized source of false-positive findings on positron emission tomography (PET) scans, typically presenting as symmetric, multifocal activity in fat-density tissue. We report a case of a 30-year-old woman with left-sided cervical swelling and unilateral, left-sided FDG uptake mimicking malignancy, but without corresponding computed tomography abnormalities. The patient's history included right-sided mediastinal ganglioneuroma resection in childhood, resulting in Horner syndrome. Hybrid PET/magnetic resonance (MR) imaging with image fusion definitively localized the FDG activity to left-sided BAT, confirming the tissue origin and linking the unilaterality to sympathetic denervation from prior right stellate ganglion disruption. This case illustrates that hybrid PET/MR can resolve diagnostic uncertainty in atypical BAT presentations and highlights the importance of recognizing altered BAT metabolism after sympathetic injury to avoid misinterpretation of PET findings.

## Introduction

Physiologic uptake of ^18^ F-fluorodeoxyglucose (FDG) in brown adipose tissue (BAT) of cancer patients is a well-described phenomenon that may confound interpretation of positron emission tomography (PET) scans, typically presenting as symmetric, multifocal uptake in low-attenuation tissue on computed tomography (CT). Here, we report a patient with left-sided cervical swelling and left-sided multifocal FDG uptake suggestive of lymphoma but without morphological correlates in corresponding CT scans that could be attributed to BAT. Hybrid PET/magnetic resonance (MRI) imaging with image fusion definitively attributed the focal FDG activity to BAT. The striking unilaterality of FDG hypermetabolism corresponded to the condition of a Horner syndrome resulting from resection of a ganglioneuroma in childhood. As BAT is almost exclusively innervated by β-3-adrenergic receptors for nonshivering thermogenesis, the unilateral metabolic activity within a bilaterally distributed BAT can be explained by unilateral deafferentiation following destruction of β-adrenergic innervation at the right stellate ganglion during tumor resection, clinically correlating with Horner syndrome.

## Case Report

**Fig. 1:**
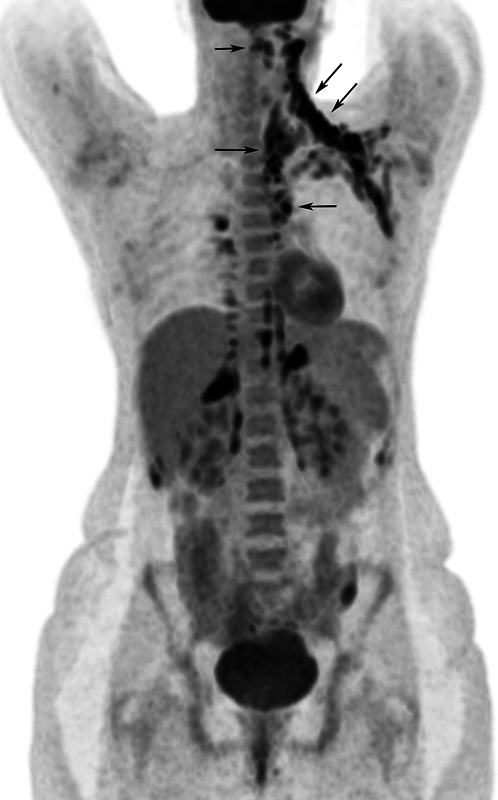
Maximum intensity projection (MIP) showing left-sided focal increased left-sided FDG uptake in cervical, supraclavicular, and thoracic sites (black arrows), mimicking possible malignant disease.

**Fig. 2:**
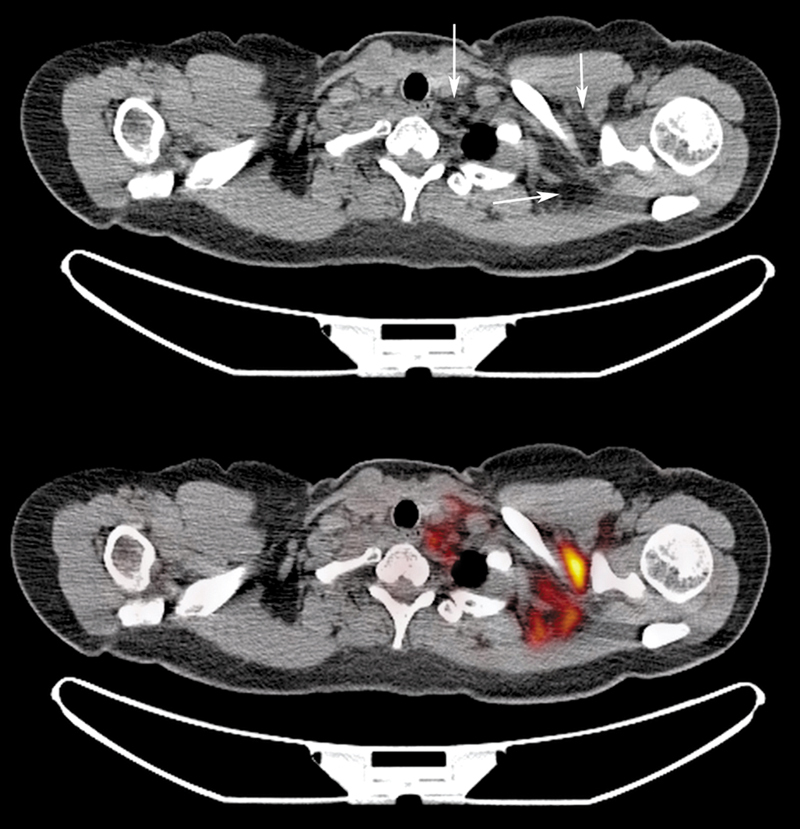
Axial slices at the level of left clavicle demonstrating low attenuating tissue without evidence of lymphonodal disease in the left supraclavicular, posterior cervical and upper left mediastinal regions (white arrows, upper row) in regions with focal FDG uptake (lower row).

**Fig. 3:**
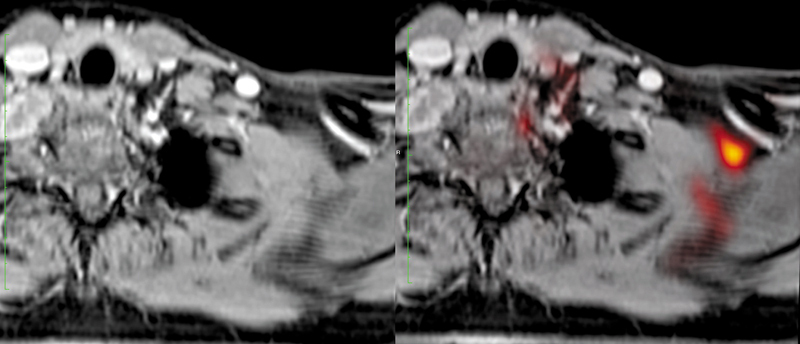
Axial gradient echo MR images (left) fused with FDG-PET/CT (right), demonstrating fat tissue at the left supraclavicular FDG uptake site (white arrow). CT, computed tomography; FDG, ^18^ F-fluorodeoxyglucose; MR, magnetic resonance; PET, positron emission tomography.

A 30-year-old woman presented with a 4-week history of left-sided cervical swelling. At age 6, she had undergone partial resection of a right posterior mediastinal ganglioneuroma, resulting in mild Horner syndrome. FDG-PET/CT was performed to exclude recurrent tumor and demonstrated exclusively left-sided, multifocal, asymmetric, and high metabolic activities in cervical, supraclavicular, and thoracic sites ( Fig. 1 ), without any morphologic correlates on the corresponding CT scans ( Fig. 2 ). No lymphonodal, focal activity was detected, nor was there evidence of tumor recurrence at the initial site of the right-sided ganglioneuroma. Apart from BAT activity, no suspicious left-sided finding explained the clinical condition. Subsequent cervical sonography and MR imaging revealed no pathological findings to explain left-sided swelling, but did demonstrate symmetrical distribution of brown fat tissue at cervical, supraclavicular, and thoracic levels. Image fusion of cervical MRI with PET (hybrid PET/MRI) confirmed localization of focal FDG activity to left-sided BAT ( Fig. 3 ).

## Discussion

FDG uptake in BAT is a phenomenon described first in 2002 1 and occurs in up to half of pediatric oncology patients undergoing PET scans, being especially common in adolescents and young patients, and more frequent during the winter season. 2 BAT is ubiquitous in vertebrates and a source of nonshivering thermogenesis. Adrenergic innervation of BAT has extensively been studied in rodents. 3 4 Beta blockers such as propranolol have shown efficacy in reducing adipose tissue uptake of FDG on PET scans in older adult oncology patients, 5 6 but may also reduce FDG uptake in tumor tissue. 7 Loss of adrenergic innervation due to sympathectomy has been shown to inhibit thermogenesis of brown fat in animal models. 8 Prior case reports demonstrating asymmetric BAT activity after surgical damage to sympathetic innervation did not include MR confirmation of BAT. 9 As thermogenesis and glucose uptake of BAT are closely correlated, 10 unilateral adrenergic deafferentiation at the level of stellate ganglion, accompanied by Horner syndrome, is the most likely explanation for the strictly unilateral BAT FDG uptake in our patient. This is the first case to demonstrate, using hybrid PET/MR and image fusion, complete inhibition of FDG uptake in human BAT after sympathectomy with definite proof of metabolically activated BAT at the same anatomical level in the same patient. Clinical knowledge of physiological and altered BAT metabolism is indispensable for correct interpretation of PET scans that appear pathologic due to asymmetric focal activity. This case demonstrates the clinical value of hybrid PET/MR imaging, where dedicated PET/MR would have provided the correct diagnosis in a single imaging session through intrinsic image correlation.
